# Therapeutic Role of Alkaloids and Alkaloid Derivatives in Cancer Management

**DOI:** 10.3390/molecules28145578

**Published:** 2023-07-22

**Authors:** Kolawole Olofinsan, Heidi Abrahamse, Blassan P. George

**Affiliations:** Laser Research Centre, Faculty of Health Sciences, University of Johannesburg, P.O. Box 17011, Doornfontein 2028, South Africa; kolawoleo@uj.ac.za (K.O.); habrahamse@uj.ac.za (H.A.)

**Keywords:** alkaloids, antineoplastic, plants, cancer therapeutics, alkaloid classifications

## Abstract

Cancer is a neoplastic disease that remains a global challenge with a reported prevalence that is increasing annually. Though existing drugs can be applied as single or combined therapies for managing this pathology, their concomitant adverse effects in human applications have led to the need to continually screen natural products for effective and alternative anticancer bioactive principles. Alkaloids are chemical molecules that, due to their structural diversity, constitute a reserve for the discovery of lead compounds with interesting pharmacological activities. Several in vitro studies and a few in vivo findings have documented various cytotoxic and antiproliferative properties of alkaloids. This review describes chaetocochin J, neopapillarine, coclaurine, reflexin A, 3,10-dibromofascaplysin and neferine, which belong to different alkaloid classes with antineoplastic properties and have been identified recently from plants. Despite their low solubility and bioavailability, plant-derived alkaloids have viable prospects as sources of viable lead antitumor agents. This potential can be achieved if more research on these chemical compounds is directed toward investigating ways of improving their delivery in an active form close to target cells, preferably with no effect on neighboring normal tissues.

## 1. Introduction

Cancer is the common name given to a group of complex pathologies triggered by factors that damage genetic materials, thus resulting in uncontrolled cell proliferation and consequent formation of abnormal cells capable of attacking other near or distant cells [1]. While genetic alterations in normal tissues can also result in benign and premalignant tumors, there are indications that major health challenges associated with cancer diseases, especially those without an early diagnosis, are significantly linked to cancerous or malignant tumors [2]. According to Sung et al. [3], cancer is the principal cause of human mortality and a limitation to long life expectancy in many developed and developing countries. With about 19.3 million people affected in 2020, deaths due to this disease amounted to 10 million out of the former estimate. Moreover, by 2040, the worldwide health challenge due to cancer is projected to increase by 47%. Cancer has the possibility of developing in nearly any tissue or organ in the body. However, as of 2020, breast (2.26 million), lung (2.21 million), prostrate (1.41 million), skin (1.19 million) and colon (1.14 million) cancers represented the leading types of this pathology documented globally [3].

Currently, various clinically approved treatment modalities exist for cancer treatment. These include chemotherapy, radiotherapy, immunotherapy, hormone therapy and targeted therapy, which may be applied alone as monotherapy or together as a combined therapy. However, despite the success recorded with these therapeutic regimes, their application in cancer management is challenged by unwanted adverse effects, such as lack of specificity or toxicity with regard to normal cells, disease re-emergence years after treatment and poor bioavailability, amongst others [4,5]. Consequently, these factors, combined with the high cost of conventional cancer therapies, have led many people, especially those in developing countries, to explore pharmacologically active chemical compounds present in plant products as alternative treatment options [6].

Plants have been described as reservoirs of bioactive compounds for cancer treatment [7]. Newman and Cragg [8] revealed that about 35% of all anticancer drugs available for cancer management between 1981 and 2014 were obtained from plant products. Alkaloids represent one of the highly diverse categories of chemical compounds in plants that regulate plant growth and protect them against herbivory [9]. They have structural diversity, which results in them possessing various pharmacological properties, including anticancer effects. While alkaloids such as vinblastine first identified from plants are now clinically approved for cancer treatment, poor solubility, low bioavailability, drug resistance and hepatic toxicity have limited experiments with some alkaloids from advancing beyond in vitro and in vivo experiments. Consequently, this article first summarizes the significance and the mode of action of various classes of new plant anticancer drugs identified between 2019 and 2022 using PubMed and the Google Scholar database. It also proposes some ways of mitigating the poor pharmacokinetic properties associated with newly identified plant-derived alkaloids.

## 2. Alkaloids and Their General Applications

Alkaloids are organic chemical compounds with a cyclic ring structure containing one or more basic nitrogen atoms. They are widely distributed in nature and are found as naturally occurring secondary metabolites in both plants and animals. Despite this distribution, screening, identification and discovery of pharmacologically relevant alkaloids with usefulness in disease management have been carried out mainly with those chemical candidates derived from plant sources [10]. While they are primarily synthesized from amino acids, alkaloids can be found in seeds, roots, stems and leaves of higher plants, such as those in the Solanaceae, Ranunculaceae, Loganiaceae, Menispermaceae, Amaryllidaceae and Papaveraceae families [11]. Although alkaloids’ function in plants is complex and not fully understood, there are indications that their production in plants could be attributed to defensive evolution against biotic factors, such as pathogens, insects and animals, that could pose threats to their existence as hosts. Chen et al. [12] showed that 7-demethoxytylophorine, a phenanthroindolizidine alkaloid, has antifungal properties, with a minimum inhibitory concentration of 1.56 µg mL^−1^ against *Penicillium italicum*, which is responsible for blue mold disease in plants [13]. Moreover, Hikal et al. [14] reviewed the insecticidal activities of quinolone, pyridine and piperidine alkaloids against pests affecting various different plants.

**Figure 1:**
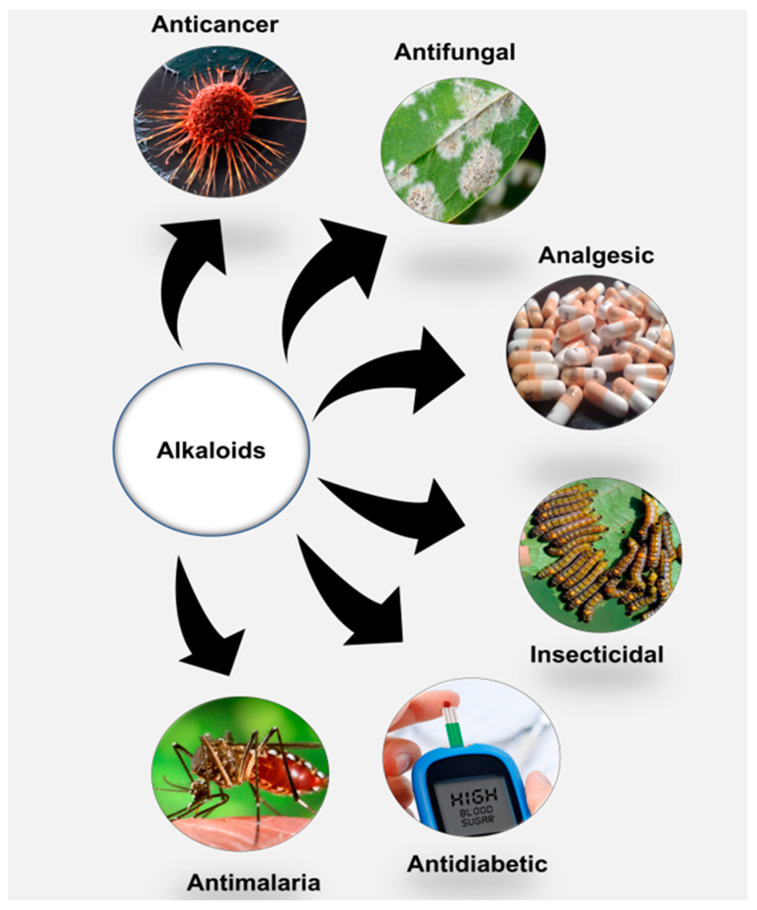
Alkaloids and their general pharmacological significance.

According to Heinrich et al. (2021), alkaloids possess some unique chemical characteristics that make them interesting candidates for use in medicine. In their basic state, they are soluble under acidic conditions, whereas they become lipid membrane-permeable when neutral after losing their protons. With these properties, alkaloids have been used in various applications in plant and human disease treatments (Figure 1). Currently, morphine and its chemical derivative codeine, obtained from *Papaver somniferu*, are often used as analgesics. Before replacement with other effective plant-derived drugs (e.g., artemisine), the antimalarial properties of quinine were well explored following the 17th century [15]. Tubocurarine and ergonovine, from claviceps and ephedra plant species, respectively, have been used to suppress bleeding due their effects in narrowing blood vessels. Alkaloids, including ephedrine and atropine, have been administered to treat respiratory illnesses, while others, such as vincristine, berberine and vinblastine, have been employed as anticancer agents [9].

## 3. Alkaloids in Cancer Treatment: A Historical Perspective

The first type of alkaloid that was applied in cancer therapy was discovered in the 1950s accidentally from Vinca rosea, a plant that is also referred to as Catharanthus roseus, by two researchers; namely, Charles Beer and Robert Noble [16]. Knowledge of the traditional use of this plant in reducing blood glucose led them to investigate its hypoglycemic effect in rats. However, instead of these antihyperglycemic properties, they discovered a considerable reduction in bone marrow damage in these animals with a simultaneous decrease in blood leucocytes and granulocytes [17]. After this finding, more focused studies on rats with lymphocytic leukemia led to the characterization of vinblastine as the first clinical anticancer use of Vinca alkaloids. To date, many other chemical compounds of the same plant category, including colchicine, vinflunine, vincamine, vinorelbine and vincristine, have also been documented for their antitumor efficacies [18,19].

## 4. Chemistry of Anticancer Alkaloids

**Figure 2:**
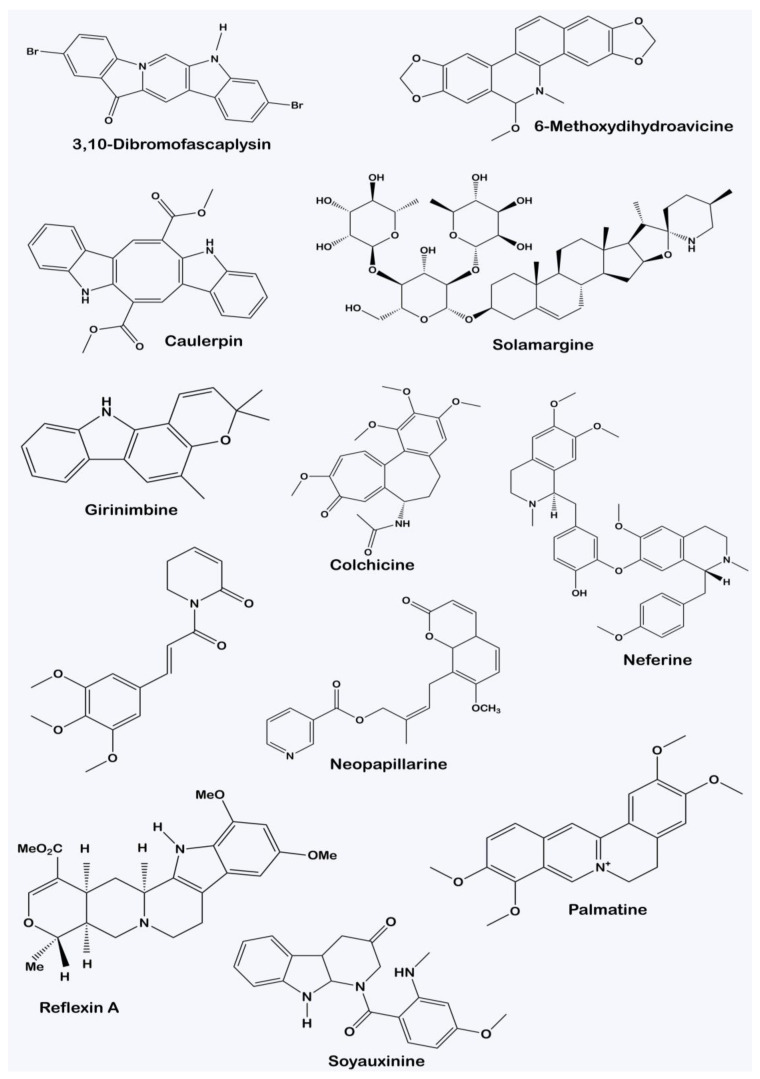
Structural diversity of alkaloids characterized from various plants.

Alkaloids are a very diverse group of plant secondary metabolites. There is no uniform pattern of classifying them due to their structural variation, as observed in Figure 2, which often does not correlate with their biological activities. For instance, alkaloids with similar ring structures but synthesized from different metabolic pathways could possess different pharmacological activities [20]. Consequently, alkaloids can be classified depending on the type of plant families they are derived from. Typical examples of this taxonomical classification include vinblastine, present in Vinca rosea, an alkaloid from the plant family Apocyanaeae; lobeline, found in *Lobelia inflata* as derived from the Lobeliaceae family; and physostigmine, present in *Physostigma venenosum* as derived from the family Leguminosae [21].

Another form of alkaloid classification is based on the precursor biomolecule used as the starting material for their biosynthetic pathways. Under this classification, alkaloids produced from the same amino acids, like arginine, tyrosine, proline, tryptophan, lysine and aspartate, can all be grouped in a class despite possessing different pharmacological activities and taxonomic distributions [22]. However, according to Dey et al. [20], the best all-inclusive classification of these basic nitrogen-containing phytochemicals involves categorizing them based on their chemical structures into tropane, pyrrolizidine, piperidine, quinoline, isoquinoline, indole, steroidal, imidazole, purine and pyrrolidine alkaloids. Although this article does not discuss all alkaloids in relation to their known biological activities, it aims to cover available information concerning anticarcinogenic drug discovery by focusing on plant-derived alkaloids documented between 2019 and 2022 as having antineoplastic properties and their mode of action.

## 5. Anticancer Alkaloids from Plants

**Figure 3:**
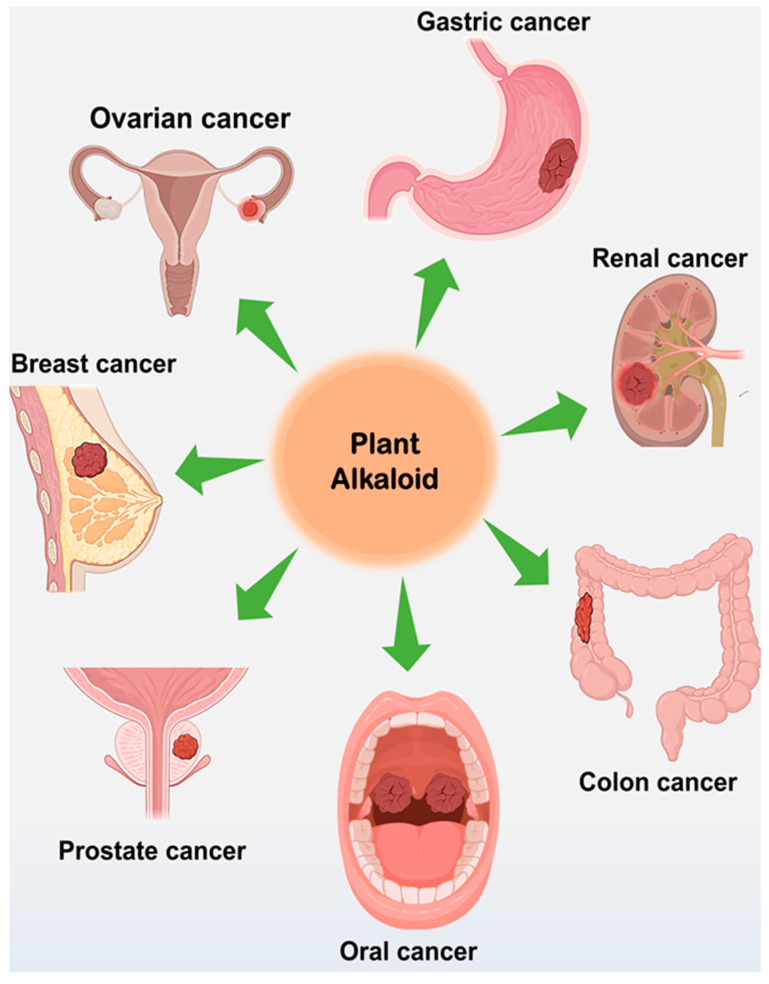
Cancer tissue targets of plant-derived alkaloids (images adapted from https://app.biorender.com/ (accessed on 14 June 2023)).

Screening of plants for pharmacologically active secondary metabolites is an ongoing process. Therefore, previously specified anticancer alkaloids identified from various plant species over 4 years are highlighted in Table 1. While these plant-derived alkaloids are discussed based on their different structural classes in the following sections, Figure 3 summarizes the various tissue targets for therapy from the positive outcomes of their in vitro anticancer experiments.

### 5.1. Proto-Alkaloids

Although alkaloids generally are described as having a basic nitrogen atom in a heterocyclic ring structure, some of these plant metabolites, known as proto-alkaloids, have nitrogen emanating from amino acid residues that is not present in different cyclic skeleton systems. They are simple alkaloids that constitute a small proportion of alkaloid phytochemicals and are often derived chemically from tryptophan, tyrosine or phenylalanine amino acids with aromatic side chains. Colchicine is a proto-alkaloid isolated from the Colchicum plant species. As a therapeutic drug, the use of colchine has been reported in the treatment of inflammatory pathologies [63]. Other studies carried out with the chemical derived from plant material suggest that it has a role in mitigating the progression of cancer diseases. According to Adham Foumani et al. [39], colchine isolated from *Colchicum autumnale* initiated apoptosis in human and mouse breast cancer cells by enhancing the cellular expression of selected caspases, tumor protein 53 (p53) and B-cell lymphoma 2-like protein 4 (Bax). The antiproliferative effect of *Colchicum pusillum* extract in colon cancer cells was associated with the alkaloid downregulation of the β-catenin-mediated signaling cascade [37]. Moreover, in another study, Sun et al. [38] indicated that treatment with 250–650 μM colchine led to oral cancer cell death via a mechanism that involved a considerable increase in cytosolic Ca^2+^ concentration due to signaling molecule release from the endoplasmic reticulum by phospholipase C activation.

### 5.2. Cumarine–Alkaloid Conjugate

Courmarin is a benzopyrone phenolic compound present naturally in many plant materials. While coumarin is vital to life processes such as photosynthesis, hormone regulation and respiration in plants, there are indications that coumarin derivatives discovered in some plant species have useful pharmacological properties. Amongst these coumarin conjugates that have been documented to have anticancer capacities is the alkaloid coumarin derivative compound neopapillarine found in the *Neocryptodiscus papillaris* plant of the family Apiaceae [50]. Investigations revealed that the coumarin alkaloid showed a preferential cytotoxic effect on renal cancer cell lines (UO31 and A498). Although this phytochemical was identified for the first time in this study, there is a need to carry out further research on this compound to unravel its cytotoxic mode of action in tumor cells.

### 5.3. Indole Alkaloids

Alkaloids with indole chemical structures constitute the largest category of alkaloid compounds. These indole parent chemical compounds contain one pentacyclic ring and a pyrrole five-membered ring with a basic nitrogen atom, reported to confer biological activity in this class of alkaloids [64]. The indole alkaloids are widely distributed among different plant families. Interestingly, clinically approved antitumor chemotherapeutic drugs, such as vinblastine and its chemical analogue vincristine, are classified as indole alkaloids. Recently, alkaloids with indole molecular structures and anticancer potential have been isolated from some plants. Fadaeinasab et al. [54] identified a new indole alkaloid called reflexin A and two other known ones from the bark of *Rauvolfia reflexa*. In vitro analysis showed that the compound invokes both early- and late-stage apoptosis in colon cancer cells. According to the authors, the early-stage apoptosis that happened in the cells was associated with caspase 9 induction, whereas the late-stage events that occurred after 48 h were linked with caspase 8 activation. Similar caspase-mediated apoptosis coupled with the inhibition of G1 phase DNA replication was reported in BEL-7402 and SMMC-7721 hepatocarcinoma cell lines treated with acetoxytabernosine alkaloid from *Alstonia yunnanensis* [30]. Another indole alkaloid identified as chaetocochin J was obtained from the fungus *Chaetomium globosum* of the plant family Chaetomiaceae. At an IC_50_ of around 0.5 μM, this compound inhibits the proliferation of SW480 and HCT116 colorectal carcinoma cells. Its anticancer ability was associated with the compound’s simultaneous elevation of the expression of phosphorylated AMP-activated protein kinase (AMPK), downregulation of phosphoinositide-3-kinase (PIK3R4) and formation of autophagolysosomes. These processes ultimately contributed to apoptosis and autophagy in the cancer cells after treatment with the compound. In the study by Dini et al. [33], another indole alkaloid, caulerpin, was isolated in addition to a phytosterol from a hexane extract of *Halimeda cylindracea* microalga collected from an Indonesian coral island. After this compound was eluted from a silica gel-packed column with n-hexane: ethanol solvent (8:2), the purified caulerpin was tested against NCL-H460 lung cancer cells. The results indicated that the compound demonstrated cytotoxic properties with an IC_50_ value of about 20.05 μg/mL.

Furthermore, investigations of caulerpin in HCT-116 and HT-29 colorectal cancer cells also suggested that the compound limited the cell migration and increased the apoptosis of the tumor cells [34]. Moreover, the insignificant toxicity of caulerpin displayed in human HDF and mouse NIH-3T3 normal fibroblast cell lines indicates its possible lesser adverse effect on normal cells if developed as an anticancer agent. In the study by Dyshlovoy et al. [25], 3,10-dibromofascaplysin, an indole alkaloid derivative, was reported to display an antiproliferative effect in 22rv1 drug-resistant prostate cancer cells. This bis-indole alkaloid, identified from *Fascaplysinopsis reticulata*, induced limited androgen receptor signaling and intensified the cells’ sensitivity to enzalutamide.

### 5.4. Quinoline Alkaloid Derivatives

The quinoline heterocyclic ring and its derivatives have been described as sources of chemical components with pharmacological usefulness in organic and medicinal chemistry [65]. More importantly, their structural scaffold has been reported as a chemical assembly for developing anticancer entities [66,67]. Quinoline is a benzopyridine organic molecule that structurally has a benzene ring fused with a nitrogen-containing pyrimidine ring. It is from this basic skeleton that various related phytochemicals are formed. Compounds with similar quinoline structures derived from plants, as presented in Table 1, include isoquinoline, benzylisoquinoline and aporphine alkaloids. While the benzylisoquinoline alkaloids are structurally related to the isoquinoline alkaloids but with a benzyl group attached to C1 of the latter, the ring system of aporphine alkaloids appears to be more complex since it has more than two fused heterocyclic structures. Despite their structural diversity, quinoline alkaloid derivatives obtained from different plant sources have been reported to show antineoplastic effects on various cancer cells. Al-Ghazzawi [29] identified two benzylisoquinolines (namely, coclaurine and 6, 7-dimethoxy-1-(α-hydroxy-4-methoxybenzyl)-2-methyl-1, 2, 3, 4-tetrahydroisoquinoline) from the sugar apple plant (*Annona squamosa*). The results obtained after testing these compounds against HCT116, MCF-7 and HEPG-2 human colon, breast and liver cancer cell lines, respectively, indicated their anticancer effects. Although the compounds had greater potency in the liver cell lines, the presence of more hydroxyl groups in coclaurine gave it superiority over the other compound. Neferine is another reported benzylisoquinoline alkaloid obtained from *Nelumbo nucifera* seed embryos [49]. The compound induced apoptosis in HeLa and SiHa cervical cancer cells by enhancing reactive oxygen species (ROS) generation while increasing the expression of cytochrome c and other apoptotic proteins. Palmatine and 6-methoxydihydroavicine isoquinoline alkaloids were isolated from *Berberis cretica* and *Macleaya cordata*, respectively [28,51]. Reports by Ma et al. [28] and Zhang et al. [27] suggest that 6-methoxydihydroavicine increased cellular ROS generation and interfered with mitochondria oxaloacetic acid metabolism during glycolysis in pancreatic and ovarian carcinoma cancer cells. In contrast, palmatine sensitizes MCF-7 cells to doxorubicin treatment by inhibiting the breast cancer estrogen receptors. However, the antiproliferative properties of the aporphine alkaloid crebanine N-oxide from *Stephania hainanensis* were due to G2/M phase cell cycle arrest and apoptosis by caspase 3 and cytochrome c protein expression [40].

Matada et al. [65] have also described the anticancer ability of the camptothecin pyrroloquinoline alkaloid, first identified from *Camptotheca acuminata*, a woody plant from China. To prevent excessive logging of this plant for the cytotoxic compound, other researchers have investigated the possibility of extracting this compound or its derivatives from endophytes cultured from the plant part. Interestingly, 10-hydroxycamptothecin, obtained from *C. acuminata* endophytic fungi (*Xylaria* sp.), has been revealed to inhibit bromodomain-containing protein 4 (BDR4) in MDA-MB-231 triple-negative breast cancer cells [31,68]. Moreover, camptothecin and its related chemicals have been reported to exert their antitumor activity by inhibiting topoisomerase 1 [32]. The complex formed when camptothecin binds to topoisomerase and DNA obstructs the movement of the replication fork and thus creates shear stress that leads to the death of the cell [32].

### 5.5. Carbazole Alkaloid

The carbazole alkaloid’s structural motif consists of a pyrrole ring with nitrogen fused at both sides by a benzene cyclic ring. This heterocyclic alkaloid class has been identified from metabolites from bacteria and plant sources. Although these alkaloids are found as natural products, their intriguing biological activities have stimulated synthetic chemists’ interest in producing them from inorganic molecules [69]. Most of the reported carbazole alkaloids found in plants have been documented in the Rutaceae family. One of the plants in this family from which several carbazole alkaloids have been characterized in the past few years is *Murraya koenigii* or the curry tree plant. Yang and Yu [45] discovered that girinimbine from this plant induced apoptosis in MDA-MB-453 breast cancer cells by inhibiting the mitogen-activated protein kinase/extracellular signal-regulated kinase (MEK/ERK) pathway, which is vital for cell survival. In another report, Satyavarapu et al. [46] isolated koenimbine, mahanimbine and mahanine from leaves of the same plant and investigated their antitumor activities against ovarian, lung and bladder cancer cell lines. In another report, mahanimbine and koenimbine lowered the metabolism of PC-3 prostate cancer and OVCAR3 ovarian cancer cells, while mahanine caused anoikis in the same cells via microtubule-associated protein (LC3) induction. This latter process, which resulted in autophagy coupled with a reduction in p*62* protein expression, was described as one of the mechanisms involved in mahanimbine-mediated cell death in Hs172.T bladder cancer cells [47].

Carbazole alkaloids have also been identified in *Glycosmis pentaphylla*. This plant of the family Rutaceae, previously known as *Glycosmis arborea*, is common to the northwest regions of Australia and southern Asia, where it is cultivated for its edible fruit [70]. In the investigation by Ito et al. [23], glybomines and other carbazole alkaloid derivatives, such as 3-methoxyl carbazole, were discovered in the acetone extract of dried *Glycosmis arborea* stem. Moreover, in a recent experiment, the characterization of the carbazole isolated from *Glycosmis pentaphylla* stem ethanol extract indicated the presence of glycosmisines A and B [71]. These compounds suppressed the proliferation of liver hepG2 and A547 lung cancer cells in a concentration-dependent manner. Glycosmisine A’s IC_50_ values for these cell lines were 50.30 μM and 43.68 μM, whereas, for glycosmisine B, the IC_50_ values were 62.89 μM and 57.10 μM. Moreover, Alanazi et al. [24] also showed that 3 methoxy carbazole mediates MCF-7 breast cancer apoptotic cell death by elevating reactive oxygen species production and caspase 3 protein expression.

Besides the Glycosmis and Murraya species, several carbazole alkaloids with anticancer bioactivities have been reported in Clausena plant species [72]. Claulansines and claulamine carbazoles were isolated from *Clausena lansiuma* stem, while clauemarazoles were present in the same morphological part of *Clausena emarginata* [73,74]. Moreover, various clausenawalline alkaloids were found to be present in acetone extract from *Clausena wallichii* twigs [75]. Andas et al. [44] purified dentatin carbazole from *Clausena excavate* root extract. The mechanism studies in this work suggested that dentatin inhibited nuclear factor kappa B (NF-κB) and increased caspase 3 and 9 expressions in HepG2 cells. In another experiment, dentatin was reported to induce apoptosis in colorectal carcinoma cells by stimulating G0/G1 cell cycle arrest while elevating the protein level of Th1-type cytokines [43].

### 5.6. Indoloquinazoline Alkaloid

Quinazoline-containing organic compounds have been described as an important pharmacologically active class of therapeutic agents. Quinazolines have a structural variant of the quinoline skeletal architecture. However, the pyrimidine cyclic ring fused with the benzene in quinazoline contains two nitrogen atoms. Indoloquinazole alkaloids are quinazoline derivatives identified from some plant species that have been reported to possess cytotoxic properties against cancer cells. One of the plants from which indoloquinazoline alkaloids have been characterized is *Araliopsis soyauxii*. In the Noulala et al. [60] study, soyauxinine and other known phytocompounds were isolated from the stem bark of the flowering plant using the nuclear magnetic resonance and mass spectroscopy analytical techniques. Treatment of CCRF-CEM cells with the compound at 3.64 μM IC_50_ altered mitochondrial membrane potential and elevated ROS levels and apoptotic caspase protein expression in the leukemia cells. However, according to Noulala et al. [61], soyauxinine showed poor antiproliferative effects on colon HT-29 and prostate PC-3 cancer cells.

### 5.7. Steroidal Alkaloid

Structurally, steroidal alkaloids are chemical derivatives of plant steroids but with one or more nitrogen atoms in their heterocyclic rings. Due to their characteristic similarities with steroids, they have been described as having the biological properties of both alkaloids and steroids [76]. With no documented role in plant development and reproduction, steroidal alkaloids are associated with protection against environmental factors that threaten plant survival [76]. Steroidal alkaloids are found in various plant species growing in Africa’s tropical and sub-tropical regions. Despite this diverse distribution and their varied biological activities, according to Abd Karim et al. [77], their presence is limited to the Buxaceae, Liliaceae, Apocynaceae and Solanaceae plant families. Solamargine is a steroidal alkaloid isolated from some Solanaceae species. Interestingly, the anticancer bioactivity of this phytocompound has been evidenced in previous studies. Solamargine evokes cell death in castration-resistant prostate cancer cells by suppressing phosphorylated Akt expression, with consequent dysfunction of the PI3K/Akt signaling pathway [58]. The compound demonstrated its antitumor properties in gastric cancer cell lines by interfering with the MAPK signaling cascade through the suppression of the nuclear paraspeckle assembly transcript 1 (NEAT1) protein level [59]. Moreover, the same compound obtained from *Solanum aculeastrum* fruits at 15.62 μg/mL IC_50_ caused a 9.1-fold inhibition of P-glycoprotein in the SH-SY5Y neuroblastoma cell line. Among the Buxaceae family plants, *Buxus sempervirens* leaves and twigs have been reported to possess cyclovirobuxine D and imperialine steroid alkaloids [78]. The formal compound, according to Lu et al. [42], induces autophagy in breast cancer cells by enhancing autophagy-related ATG5 expression, converting autophagosome biomarker LC3 from type I to III and concomitantly suppressing the Akt/mTOR signaling pathway. Paravallarine is another sterol alkaloid with anticancer properties. In the experiment in [79], this alkaloid was obtained from *Kibatalia laurifolia* (Apocynaceae) leaves with some other compounds. Moreover, cyclopamine isolated from *Veratrum californicum* in the Liliaceae family was documented to induce apoptosis of cancerous breast cells through the distortion of Smo protein function by impeding Hedgehog signaling cascade [41,80]

### 5.8. Piperidine Alkaloid

Piperidine alkaloids have a piperidine heterocyclic chemical structure with one amide bond and five methylene linkages. Although piperidine alkaloids such as euphococcinine and pinidinone have been identified in insects, the majority of these alkaloids investigated for the use of their lead pharmacological properties in drug development are from plants [81]. More importantly, studies have described plant species in the Piperaceae family as rich sources of piperidine alkaloids [82]. One of these plants is *Piper nigrum* or black pepper, which has been described as a source of essential piperidine alkaloids, including piperidine and its substituted derivatives. Interestingly, these two compounds have been widely researched for their antitumor activities against different cancer cells [83,84]. Piperlongumine is another piperidine alkaloid isolated from *Piper longum* or Indian long pepper, which belongs to the Piperaceae plant family. Awasthee et al. [52] evaluated the anticancer effects of this alkaloid on different cancer cell lines (MCF-7, MDA-MB-468, T-47D, MDA-MB-231) as a single treatment and in combination with doxorubicin. The results showed that the alkaloid at 1–20 μM dose-dependently lowered glucose uptake by modulating glucose transporter-1 (GLUT-1) with concomitant elevation of monocarboxylate transporter 4 (MCT-4) expression in the breast tumor cells. Furthermore, the alkaloid at 20 μM potentiated doxorubicin-mediated cytotoxicity in the cell by 40%. In another experiment with a Balb/c mice in vivo model, piperlongumine suppressed Bcl-2 protein level and inhibited the G2/M phase of the cell cycle in colonocytes isolated from animals with 1,2-dimethylhydrazine/dextran sulphate sodium-induced colon carcinogenesis [53].

*Piper methysticum* is another Piperaceae reported to possess piperidine alkaloids. In Dragull et al.’s [85] study, two major piperidines, awaine and pipermethystine, were obtained from various parts of this plant. While the first was found in the young leaves, the second was more concentrated in the peels of the plant stems. Surprisingly, piperidine alkaloids have also been documented in plants that do not belong to Piperaceae. The report by Viegas et al. [86] described the isolation of four piperidine alkaloids from *Cassia spectabilis* of the Fabaceae plant family. These compounds included iso-6-spectaline, 7-hydroxyspectaline, spectaline and 3-O-acetylspectaline. While the authors found these alkaloids in the flowers, they discovered that the latter two were also present in the green fruits of the plant. *Microcos paniculate* (Malvaceae) leaves have also been reported to contain microcosamine A and C piperidine alkaloids [87]. Interestingly, studies by Still et al. [48] revealed that microcosamine A displayed antiproliferative effects in HT-29 colon cancer cells, acting as an antagonist of nicotinic acetylcholine receptors (α4β2 and α3β4).

## 6. Plant Alkaloids in Clinical Trials

Many anticancer alkaloids first identified from plant sources, such as vincristine and vinblastine, have passed rigorous drug screening tests, including human trials, prior to their approval by government agencies as clinical therapeutic drugs. Interestingly, knowledge of structure–activity relationships has also encouraged chemists to synthesize various analogs of these compounds to formulate better drugs with enhanced potency [88]. However, the associated toxicity linked with these synthetic antitumor products has resulted in the need to discover more phytoproducts with lower adverse effects for human disease management. Tilaoui et al. [89] described some clinical experimental work on some known plant-derived alkaloids in relation to cancer treatment. Amongst these anticancer alkaloids was homoharringtonine, obtained from the *Cephalotaxus fortunei* plant. Homoharringtonine, a protein synthesis inhibitor approved by the US Food and Drug Administration (FDA), resulted in a hematologic recovery rate of about 72% in patients with severe myeloid leukemia [90,91]. In a more recent randomized investigation with children (>2 years) with this same disease, the authors of [92] suggested that homoharringtonine resulted in a superior 88.0% ± 6.5 5-year event-free survival rate compared to 60.2% ± 9.6% in children managed with an anthracycline treatment regimen. Since P65 activation is involved in MYC gene overexpression in blood malignancies, homoharringtonine downregulates this process by causing P-65 to bond tightly with NF-κB repressing factor (NKRF) [93,94].

Although caffeine and its related plant-derived alkaloid theobromine are well known for their diverse pharmacological properties, their potential chemo-preventive effects in breast cancer have also been reported [95]. Before undergoing tumor operation, patients were fed capsules containing 19.7 mg caffeine/theobromine and a 473.7 mg mixture of 37 phenolic compounds. Analysis of malignant tissue metabolites showed the presence of theobromine amongst other compounds. Although methylxanthine did not exert a cytotoxic effect in MCF-7 breast cancer cells at the concentration at which it was detected in the malignant tissue, the authors suggested that the persistence of these metabolites despite fasting before surgery at a level comparable with normal subjects may necessitate a further clinical investigation of their long-term effect in cancer management.

Another plant alkaloid studied in human experiments in the last 5 years is berberine. In Chen et al.’s [96] study, this isoquinoline alkaloid was given twice daily (0.3 g) to 553 individuals aged 18–75 years who had undergone complete polypectomy after being verified to have colorectal adenoma. Six months after the patients’ surgery, treatment with berberine was carried out for 2 years before reoccurrence of colorectal adenomas in the subjects was evaluated. Besides complaints about slight constipation from the participants, the drug was found to prevent colorectal cancer reappearance at the experimental dose without any serious adverse effects.

## 7. Limitations of Alkaloids in Cancer Treatment

Despite the significant contributions of alkaloids and their various derivatives in cancer disease therapeutics, the complete adoption of these diverse biologically active nitrogen-containing heterocyclic compounds is still limited by several ongoing pharmacological challenges. One of these problems is the issue of bioavailability. Before a drug ingested or injected into the body can exert a therapeutic effect in its target tissue, it must penetrate the site in active form and at a specific effective concentration. However, alkaloids from plants generally have low bioavailability, which studies have associated with their low solubility in body fluids with poor cell membrane permeability [97]. To enhance alkaloids’ transport through the body’s aqueous system and their effective concentration at their intended site of action, Sindhoor et al. [98] suggested their inclusion in organic carriers, such as liposomes, dendrimers or polymer-based formulations. Liposome molecules such as phosphatidylcholine can hide hydrophobic chemical compounds in their core while exposing their hydrophilic end to the exterior in contact with polar body fluids. They can help transport alkaloid drugs through the blood to various target tissues in this form. Interestingly, the specificity of alkaloid-carrying liposomes can be enhanced by conjugating them with other components that can be identified by their proteins, such as folate receptors, which are highly expressed on the surfaces of many tumor cells. Previous findings have shown the anticancer efficacy of doxorubicin-liposome carrier increased significantly after surface functionalization with polyethylene glycol [99,100].

Chemotherapy is a major cancer treatment method but the success achieved using this procedure over time has been limited by the problem of drug resistance. Some cancer cells have devised ways of evading death from certain drug treatments by altering some endogenous protein isoforms, increasing the cellular expression of specific efflux proteins and repressing apoptotic event pathways [17]. These mechanisms, amongst other processes, were reported in a study of lung cancer cells’ resistance to Vinca alkaloid treatment [17]. The results of the reverse transcriptase polymerase chain reaction analysis carried out on tumor samples obtained from patients with non-small cell lung cancer revealed a considerable increase in p-glycoprotein expression during chemotherapy treatment, including vincristine and vinorelbine alkaloids [101]. Vinca alkaloids are substrates for p-glycoproteins [102]. Consequently, these phytocompounds’ efficacies can be enhanced by combining them with other chemotherapeutic drugs.

Regardless of the different antiviral, antidiabetic and antibacterial biological activities of some pyrrolizidine alkaloids [103], one of the main constraints that has prevented their screening for possible anticancer activities beyond in vitro and in vivo small animal experiments is their potential toxicity after metabolism in humans. Findings have shown that pyrrolizidine alkaloids in the liver are oxidized at C1 and C2 by the cytochrome P450 system to produce a dehydrogenated derivative with a pyrrole ring system after ingestion. This structure then spontaneously releases the oxygenated group linked to C7 and C9 of the core heterocyclic motif to produce a chemically active species capable of reacting with protein and genetic macromolecules [104]. Due to this genotoxic effect, pyrrolizidine alkaloids could provide an interesting source of lead antitumor compounds. However, their application in practical cancer therapy may not be realistic because of the adverse impact of their active metabolite on normal cells. Nevertheless, using a gold-based artificial metalloenzyme instead of the P450 liver enzyme as a catalyst, Kurimoto et al. [105], in their studies, described the conversion of a cyclized dehydrogenated pyrrolizidine precursor to an active form that produced significant cytotoxicity in HeLa, A549 and SW620 cancer cell lines. This experiment showed that, in the absence of liver metabolism, pyrrolizidine can be an effective anticancer agent without a negative effect on normal cells if artificially converted into its active form near the target tissue.

## 8. Conclusions 

Due to changing lifestyles and increasing industrialization, the prevalence of cancer has been projected to rise in the coming years. Despite the effectiveness of the available treatments for this pathology, the adverse outcomes of existing anticancer drugs require a continuous search for alternative therapeutic chemicals, especially from plant sources. In this regard, phytochemicals of natural origin, collectively described as alkaloids, have proven to be viable sources of potent antitumor compounds. There is a need to discover more of these natural products, that which provide better activities with lower toxicities in relation to the presently approved synthetic anticancer drugs. While they may possess low solubility and bioavailability, more research should be carried out investigating the various delivery methods that can be employed to improve the solubility of the already identified plant-derived alkaloids while also determining ways of enhancing their other pharmacokinetic properties to enable practical application in cancer treatment. As most of the available studies on these plant alkaloids were undertaken in vitro, a better understanding of these compounds’ mechanism of action can be obtained if they are subject to in vivo investigations with small animals. These findings will allow researchers to identify more alkaloids that could advance in testing in pre-clinical and clinical studies.

## Figures and Tables

**Table 1:** Anticancer alkaloids identified form different plant species.

Compound	Structural Classification	Plant Source	Plant Family	Mode of Action	References
3-Methoxy carbazole	Carbazole alkaloid	*Glycosmis arborea*	Rutaceae	Increased reactive oxygen species production and caspase 3 protein expression in MCF-7 cells	[23,24]
3,10-Dibromofascaplysin	Indole alkaloid	*Fascaplysinopsis reticulata*	Thorectidae	Inhibition of androgen receptor signaling Activation of C-jun N-terminal kinase (JNK) in prostate cancer cells	[25,26]
6-Methoxydihydroavicine	Isoquinoline alkaloids	*Macleaya cordata*	Papaveraceae	Activation of receptor-interacting serine/threonine protein kinase 1 and alteration of oxaloacetic acid metabolism and pancreatic cancer cells Stimulation of reactive oxygen species activation of mitogen-activated protein kinase pathway in ovarian cancer cells	[27,28]
6, 7-Dimethoxy-1-(α-hydroxy-4-methoxybenzyl)-2-methyl-1, 2, 3, 4-tetrahydroisoquinoline	Benzylisoquinoline alkaloid	*Annona squamosa*	Annonaceae	NA	[29]
Acetoxytabernosine	Indole alkaloid	*Alstonia * *yunnanensis*	Apocynaceae	Promotion of caspase 3- and caspase 9-mediated apoptosis in hepatocellular carcinoma cells	[30]
Camptothecin	Pyrroloquinoline alakloid	*Camptotheca * *acuminata*	Nyssaceae	Inhibition of topoisomerase 1 and BRD4 in MDA-MB-231 breast cancer cells	[31,32]
Caulerpin	Indole alkaloid	*Halimeda * *cylindracea,* *Halimeda * *lentillifera*	Halimedaceae	Enhancement of cell migration and induction of apoptosis in colorectal cancer cells	[33,34]
Chaetocochin J	Indole alkaloid	*Chaetomium * *globosum*	Chaetomiaceae	Induction of autophagy via activation of PI3K/AKT/mTOR pathway in colorectal cancer cells	[35,36]
Coclaurine	Benzylisoquinoline alkaloid	*Annona squamosa*	Annonaceae	NA	[29]
Colchicine	Proto-alkaloid	*Colchicum * *pusillum* *Colchicum * *autumnale*	Colchicaceae Liliaceae	Induction of apoptosis by increasing P53, BAX and caspase 3 and 9 protein expression in breast cancer cells Significant increase in cytosolic Ca^2+^ concentration by activation of phospholipase C in oral cancer cells	[37,38,39]
Crebanine N-oxide	Aporphine alkaloid	*Stephania * *hainanensis*	Menispermaceae	G_2_ phase cell cycle arrest Increase in the expression of cytochrome c and caspase 3 in gastric cancer cells	[40]
Cyclopamine	Steroidal alkaloid	*Veratrum * *californicum*	Liliaceae	Inhibition of hedgehog signaling cascade by altering Smo protein function	[41]
Cyclovirobuxine D	Steroidal alkaloid	*Buxus * *sempervirens*	Buxaceae	Increase in ATG5 protein expression and suppression of Akt/mTOR signaling pathway in breast cancer cells	[42]
Dentatin	Carbazole alkaloid	*Clausena excavate*	Rutaceae	Elevation of Th1 cytokine protein expression Suppression of NF-κB and activation of caspase 3 and 9 in HepG2 cells	[43,44]
Girinimbine	Carbazole alkaloid	*Murraya koenigii*	Rutaceae	Inhibition of MEK/ERK pathway Downregulation of Bcl-2 in breast cancer cells	[45]
Koenimbine	Carbazole alkaloid	*Murraya koenigii*	Rutaceae	Disruption of energy metabolism in prostate cancer cells	[46]
Mahanimbine	Carbazole alkaloid	*Murraya koenigii*	Rutaceae	Arrest of G0/G1 phase cell cycle through reduction in cyclin E and cyclin D1-D3 expression in bladder cancer cells	[46,47]
Mahanine	Carbazole alkaloid	*Murraya koenigii*	Rutaceae	Oxidative stress-mediated activation of LC3 proteins Reduction in p62 expression in ovarian cancer cells	[46]
Microcosamine A	Piperidine alkaloids	*Microcos * *paniculate*	Malvaceae	Inhibition of nicotinic acetylcholine receptors in colon cancer cells	[48]
Neferine	Benzylisoquinoline alkaloid	*Nelumbo nucifera*	Nelumbonaceae	Increase in cellular generation of ROS and elevated expression of cytochrome c in cervical cancer cells	[49]
Neopapillarine	Cumarino alkaloid	*Neocryptodiscus papillaris*	Apiaceae	NA	[50]
Palmatine	Isoquinoline alkaloids	*Berberis cretica*	Berberidaceae	Suppression of estrogen receptor signaling in breast cancer cells	[51]
Piperlongumine	Piperidine alkaloid	*Piper longum*	Piperaceae	Inhibition of glucose transport and TNF-α-induced NF-κB activation in breast cancer cells Reduction in Bcl-2 expression in colonocytes of rats with colon cancer	[52,53]
Reflexin A	Indole alkaloid	*Rauvolfia reflexa*	Apocynaceae	Induction of G_1_ cell cycle arrest Activation of apoptotic caspases in colon cancer cells	[54]
Solamargine	Steroidal alkaloid	*Solanum * *aculeastrum* *Solanum nigrum*	Solanaceae	Interference with PI3K/Akt signaling in prostate carcinoma Reduction in NEAT1 protein levels in gastric cancer cells Inhibition of P-glycoprotein in neuroblastoma cells	[55,56,57,58,59]
Soyauxinine	Indoloquinazoline alkaloid	*Araliopsis * *soyauxii*	Rutaceae	Alteration of mitochondria membrane polarization and increased ROS production in leukemia cells	[60,61,62]

NA = Not available in the cited article.

## Data Availability

Not applicable.
